# Head Growth and Fundoscopy as Proxies for Intracranial Pressure in Metopic Synostosis Treated Surgically vs Conservatively

**DOI:** 10.1001/jamanetworkopen.2025.59871

**Published:** 2026-02-24

**Authors:** Pauline A. E. Tio, Evi N. Koehoorn, Lauren I. F. Clement, Dimitris Rizopoulos, Sjoukje E. Loudon, Marie-Lise C. van Veelen, Jochem K. H. Spoor, Sarah L. Versnel, Mieke M. Pleumeekers, Irene M. J. Mathijssen

**Affiliations:** 1 Department of Plastic and Reconstructive Surgery, Erasmus University Medical Center, Rotterdam, the Netherlands; 2 Department of Biostatistics, Erasmus University Medical Center, Rotterdam, the Netherlands; 3 Department of Ophthalmology, Erasmus University Medical Center, Rotterdam, the Netherlands; 4 Department of Neurosurgery, Erasmus University Medical Center, Rotterdam, the Netherlands

## Abstract

**Importance:**

The risk of increased intracranial pressure (ICP) in metopic synostosis remains poorly defined, leading to uncertainty about the need for routine surgical intervention vs conservative management.

**Objective:**

To compare head growth trajectories and the prevalence of signs of increased ICP among patients with metopic synostosis managed surgically or conservatively.

**Design, Setting, and Participants:**

This prospective cohort study included all 209 new patients with single-suture metopic synostosis presenting to Erasmus Medical Center, Rotterdam, the Netherlands, from January 1, 2017, to December 31, 2024. The current analysis included data collection up to July 31, 2025. Patients underwent annual follow-up with head circumference measurement and fundoscopy.

**Intervention:**

Surgical or conservative management of metopic synostosis determined through shared decision-making between clinicians and parents.

**Main Outcome and Measures:**

The primary outcome was longitudinal head circumference standard deviation (SD), analyzed using linear mixed models. Secondary outcomes were head growth deflection and papilledema, understood as signs of increased ICP, detected on fundoscopy.

**Results:**

Among 209 patients (78 surgical and 131 conservative; median age at presentation, 4 months [IQR, 2-7 months]; 154 boys [74%]), the median age at last follow-up was 40 months (IQR, 24-61 months). Head circumference SD increased significantly with age in a nonlinear pattern (age spline *df* = 1; β = 0.87; 95% CI, 0.54-1.21; *P* < .001; and age spline *df* = 2; β = 0.73; 95% CI, 0.29-1.16; *P* < .001). Female sex was associated with higher SD (β = 0.31; 95% CI, 0.03-0.60; *P* = .03). Surgical treatment was associated with higher initial SD but a significantly flatter growth trajectory over time compared with conservative treatment (β = –1.02; 95% CI, –1.52 to –0.52; *P* < .001 for interaction age spline and treatment). Severe deformity phenotype was associated with lower SD values compared with mild deformity phenotype (β = –0.39; 95% CI, –0.75 to –0.04; *P* = .03). Among 158 patients with 2 or more years of follow-up, head growth deflection occurred among 3 patients (1.9%): 2 of 65 (3.1%) in the surgical group and 1 of 93 (1.1%) in the conservative group (*P* = .57). Papilledema was observed in 3 patients (1.4%): 2 of 78 (2.6%) in the surgical group and 1 of 131 (0.8%) in the conservative group (*P* = .56). None of these patients required additional surgery.

**Conclusions and Relevance:**

In this prospective cohort of patients with metopic synostosis, signs of elevated ICP were rare and did not differ between surgical and conservative groups. Although surgery altered head growth trajectories, the low prevalence of ICP-related findings supports close monitoring and selective surgical intervention as safe alternatives to routine surgery.

## Key Points

**Question:**

What are the head circumference trajectories and prevalence of signs of increased intracranial pressure (ICP) among patients with metopic synostosis treated conservatively vs surgically?

**Findings:**

In this cohort study of 209 patients, surgically treated patients showed earlier plateauing of head growth, while conservatively treated patients demonstrated steady head circumference increases over time. Signs of increased ICP were rare (1.4%-1.9%) and did not differ between the 2 groups

**Meaning:**

These findings suggest that routine surgery may not be necessary to prevent ICP for most patients with metopic synostosis, supporting close monitoring and individualized treatment decisions.

## Teaser

This cohort study compares head growth trajectories and the prevalence of signs of increased intracranial pressure among patients with metopic synostosis managed surgically vs conservatively.

## Introduction

Metopic synostosis, also known as trigonocephaly, is the second most common form of craniosynostosis, occurring in approximately 1 of 4500 live births.^1^ It results from premature fusion of the metopic suture and is characterized by a triangular-shaped forehead, hypotelorism, and bitemporal narrowing.^2,3^ In addition to cranial deformity, affected children are at increased risk of ophthalmologic problems, including refractive errors and amblyopia, as well as neurocognitive and behavioral problems.^4,5,6,7,8,9,10^

Traditionally, surgical intervention has been advocated both to correct cranial deformities and to prevent secondary complications, particularly increased intracranial pressure (ICP).^11^ The risk of increased ICP in metopic synostosis, however, is less well established than in other types of craniosynostosis. The reported prevalence ranges widely, from 0% to 33%, depending on diagnostic methods and study design.^12,13,14,15,16,17^ Because the fastest phase of brain growth occurs in the first 2 years of life, this period is considered most vulnerable to increased ICP. Criterion standard assessment with invasive ICP measurement is rarely justified for otherwise healthy children, leaving clinicians reliant on noninvasive proxies, such as head growth and fundoscopy.^18,19^ Both methods, despite limitations, are practical tools for longitudinal surveillance in craniosynostosis care.^12,20,21,22^

Conservative management has gained interest as an alternative to surgery, especially for patients with mild to moderate severity of trigonocephaly.^23,24^ Avoiding surgery reduces perioperative risk and family burden, but evidence on safety remains limited. Only small cohorts have suggested that conservatively managed patients rarely require delayed intervention for ICP-related concerns.^13^ To date, no prospective studies have systematically compared head growth trajectories and ICP-related outcomes between patients treated surgically and those treated conservatively.

To our knowledge, this is the first prospective cohort study to longitudinally compare head growth and signs of increased ICP in both surgically and conservatively managed patients with metopic synostosis. By focusing on the early developmental years and using standardized, noninvasive monitoring, this study aims to provide evidence to guide individualized treatment decisions and to establish the role of conservative management in routine clinical care of metopic synostosis.

## Methods

### Design

This study is part of an ongoing prospective cohort study at the Dutch Craniofacial Center, Sophia Children’s Hospital (Erasmus Medical Center, Rotterdam, the Netherlands), registered at ClinicalTrials.gov (NCT06069479). The study protocol has been described previously.^25^ The study has been approved by the Erasmus Medical Center medical ethics committee. The Strengthening the Reporting of Observational Studies in Epidemiology (STROBE) reporting guideline was followed. Written informed consent was obtained from parents prior to participation.^25^ The Dutch Craniofacial Center serves as the largest of 2 national referral centers for craniosynostosis, ensuring that findings are generalizable to the national population and not biased by regional differences.

### Participants

All new patients with metopic synostosis presenting at our clinic between January 1, 2017 and December 31, 2024, were eligible. Exclusion criteria included prior craniofacial surgery at another center or multisuture craniosynostosis. Syndromic cases were included, and data on the type of syndrome were collected. Eligible patients were required to have at least multiple head circumference measurements during 2 or more years of follow-up and/or at least 1 fundoscopic examination. This is part of an ongoing prospective study. The data used for this analysis were collected up to July 31, 2025. Patients and their parents were informed about the study by their clinician and, if interested, were provided detailed information by an independent researcher (P.A.E.T.).

### Treatment and Follow-Up

Since 2017, parents of children with metopic synostosis have been offered the choice between conservative or surgical management at Sophia Children’s Hospital. At diagnosis, clinicians provide the following information to all parents: (1) all children are monitored identically, with surgery offered later if signs of ICP develop among those in the conservative group; (2) there is no evidence that surgery is medically necessary to prevent increased ICP; and (3) the long-term natural evolution of cranial shape without surgery is incompletely known, although some spontaneous improvement is expected. Conservative treatment involves a nonsurgical approach with yearly routine follow-up appointments. The choice of the type of surgical treatment depends on the age at presentation and parental preferences, with two options available: fronto-orbital advancement and endoscopic-strip craniectomy with helmet therapy. At our center, fronto-orbital advancement is preferably performed between 9 and 12 months of age and endoscopic-strip craniectomy between 3 and 6 months of age. If parents opt for a conservative policy, surgery is performed only if increased ICP occurs. Irrespective of treatment type, all patients have identical follow-up care, which includes yearly follow-up appointments up to 8 years of age.

### Outcomes

The primary outcome was head growth, defined as longitudinal head circumference measurements, as head circumference reliably correlated with intracranial volume.^21,26^ A deflection or stagnation in head growth is a proxy indicator for elevated ICP in patients with craniosynostosis.^12,27^ Head circumference measurements were obtained manually with a measuring tape in the axial plane from the occiput to the forehead by trained clinicians (S.L.V., M.M.P., and I.M.J.M.), expressed in centimeters, and converted to age- and sex-adjusted standard deviation (SD) scores using Dutch population norms.^28^ In our clinic, this measurement is performed yearly from initial presentation up until the age of 8 years.

The secondary outcomes are proxies for increased ICP and include the presence or absence of both head growth deflection and papilledema. A decrease in head growth of more than 0.5 SD over 2 years was prespecified as clinically relevant, based on prior craniosynostosis research.^12,21,27^ Head growth deflection was dichotomized as present or absent. For this specific outcome, only patients with head circumference measurements available over a follow-up period of at least 2 years were included in the analysis. Fundoscopy was performed annually by a pediatric ophthalmologist (S.E.L.) between 1 and 4 years of age. Papilledema was dichotomized as present or absent, with pseudopapilledema excluded after ophthalmologic and orthoptic evaluation.

The initial severity of the metopic synostosis was scored using the suture-specific metopic photo score, a validated 4-point scale (normal, mild, moderate, and severe) based on standardized 2-dimensional photographs.^29^ This severity measure was validated on a group of 26 expert craniofacial plastic surgeons and neurosurgeons from 6 European expertise centers, with the best agreement on the overall phenotype. For this study, 2 craniofacial plastic surgeons (M.M.P. and I.M.J.M.) with at least 4 years of experience were asked to score all patients. The interrater reliability showed strong agreement between the 2 independent raters (κ coefficient, 0.83; 95% CI, 0.78-0.88).

### Statistical Analysis

R, version 4.4.1 (R Project for Statistical Computing) was used for the statistical analysis. Categorical variables were described with frequencies and percentages, and continuous variables were described with mean (SD) or median (IQR) values. A linear mixed model was fitted to assess the longitudinal development of head circumference SD among patients with metopic synostosis, with age modeled as a natural spline (*df* = 2) and interaction terms included for treatment. A natural spline with 2 *df* was chosen to flexibly model nonlinear age effects while avoiding overfitting. The final model accounted for fixed effects of age, sex, treatment type (surgical vs conservative), initial severity (photo score), and interactions between treatment and age. Other interaction terms did not improve the model fit. Age was used as the random effect. Model assumptions were validated using plots to check for normality and homoscedasticity of residuals. Categorical variables were analyzed using χ^2^ or Fisher exact tests, as appropriate. Continuous variables were analyzed using *t* tests or Wilcoxon rank sum tests, depending on normality. Logistic regression analysis for the presence of papilledema was not appropriate given the low number of events. Missing data were not imputed. All available data points were included in the analysis, allowing for an unbalanced longitudinal dataset. Patients with missing measurements at specific time points were retained in the model using available case analysis. *P* < .05 was considered significant.

## Results

### Participant Characteristics

**Figure 1.**
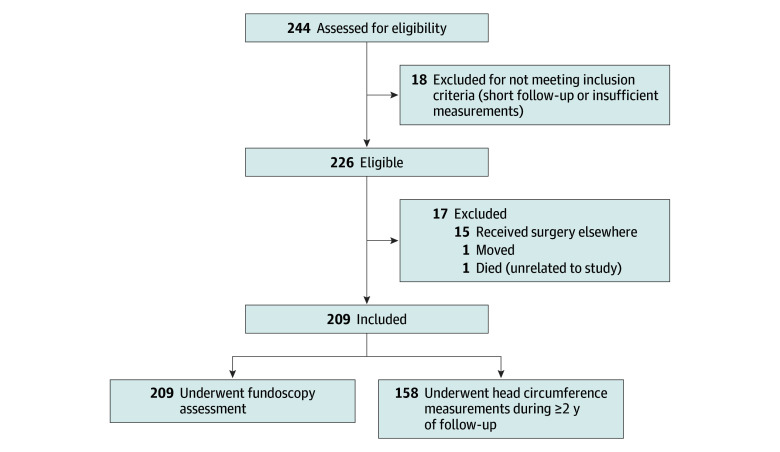
Patient Inclusion Diagram

Figure 1 presents the flow diagram of study participants. Of 226 patients eligible for inclusion, 17 were excluded, and 209 patients with metopic synostosis (median age at presentation, 4 months [IQR, 2-7 months]; 154 boys [74%] and 55 girls [26%]) were included in the analysis (Table 1). Of these, 78 (54 boys [69%] and 24 girls [31%]) underwent surgical treatment, and 131 (100 boys [76%] and 31 girls [24%]) were managed conservatively. Baseline characteristics are summarized in Table 1. The median age at the time of surgery for all patients who opted for a surgical intervention was 9 months (IQR, 4-10 months). The median age was 9 months (IQR, 9-11 months) at the time of fronto-orbital advancement and 3 months (IQR, 3-4 months) at the time of endoscopic-strip craniectomy. The median age at last follow-up for the total cohort was 40 months (IQR, 24-61 months). No patients in the conservative group required surgery during follow-up for increased ICP, and no parents asked for surgery for aesthetic concerns.

**Table 1.** Baseline Demographic and Clinical Characteristics of Patients

Characteristic	All patients (N = 209)	Conservatively treated patients (n = 131)	Surgically treated patients (n = 78)
Sex, No. (%)			
Female	55 (26)	31 (24)	24 (31)
Male	154 (74)	100 (76)	54 (69)
Pathogenic variant or syndrome present, No. (%)	19 (9)	7 (5)	12 (15)
Preoperative severity, No. (%)			
Mild	73 (35)	58 (44)	15 (19)
Moderate	89 (43)	55 (42)	34 (44)
Severe	47 (22)	18 (14)	29 (37)
Surgery type, No.			
Fronto-orbital advancement	NA	NA	49
Endoscopic-strip craniectomy	NA	NA	29
Age at surgery, median (IQR), mo	NA	NA	9 (4-10)
Age at last follow-up, median (IQR), mo	40 (24-61)	37 (24-55)	51 (25-70)

Abbreviation: NA, not applicable.

### Head Growth Trajectories

**Figure 2.**
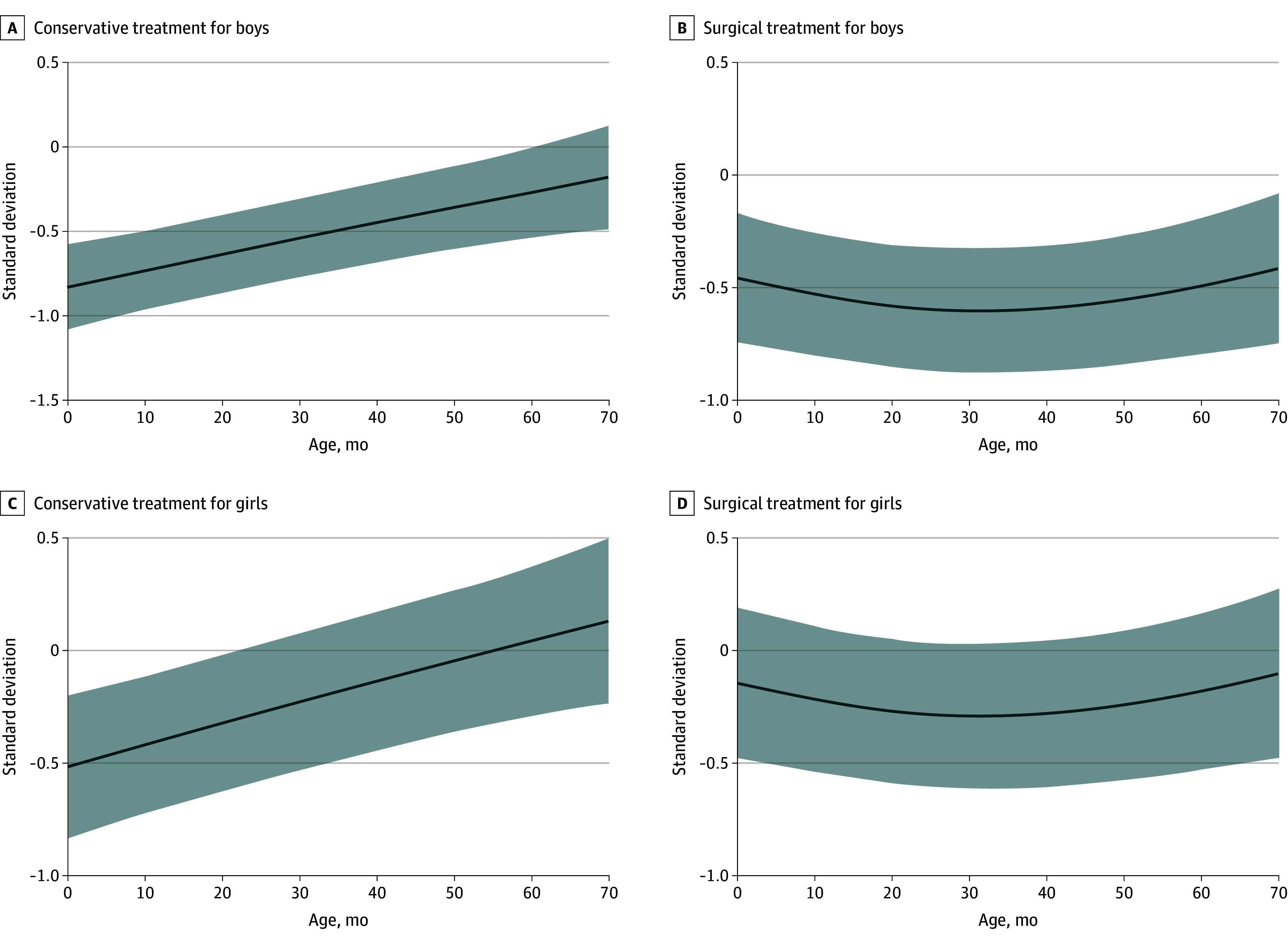
Line Plots of the Linear Mixed Model for Head Circumference Standard Deviation Over Time for the Conservative and Surgical Group, Divided by Sex The solid lines represent the model-estimated trajectories, and the shaded areas represent 95% CIs.

**Figure 3.**
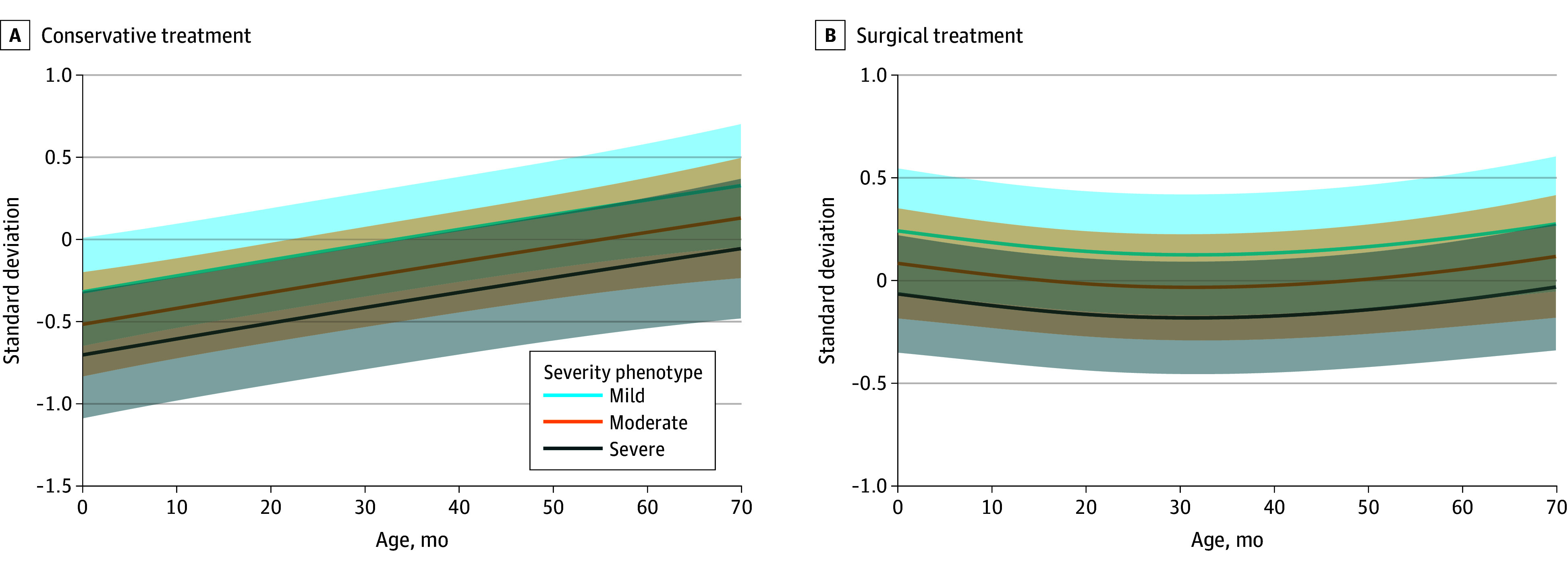
Line Plots of the Linear Mixed Model for Head Circumference Standard Deviation Over Time for the Conservative and Surgical Group, Divided by Phenotype The solid lines represent the model-estimated trajectories, and the shaded areas represent 95% CIs.

Figure 2 illustrates head circumference SD trajectories over time, stratified by treatment type and sex, as estimated by the linear mixed model. Overall, head circumference SD followed a nonlinear trajectory with age, differing significantly between treatment groups (β = 0.37; 95% CI, 0.06-0.71; *P* = .02). In the conservative group, SD increased steadily over time, while the surgical group initially showed higher SD values, which plateaued after approximately 20 months of age (β = –1.02; 95% CI, –1.52 to –0.52; *P* < .001 for interaction age spline and treatment). Figure 3 illustrates the estimated head circumference trajectories stratified by severity and treatment.

Female patients had significantly higher SD values than male patients (β = 0.31; 95% CI, 0.03-0.60; *P* = .03), consistent across treatment groups. Patients with severe deformities had significantly lower SD values than those with mild deformities (β = –0.39; 95% CI, –0.75 to –0.04; *P* = .03), while differences for moderate vs mild deformities were not statistically significant (β = –0.22; 95% CI, –0.50 to 0.07; *P* = .15). Full model results are presented in the eTable in Supplement 1.

### Head Growth Deflection and Papilledema

Among 158 patients with 2 or more years of follow-up (93 in the conservative group and 65 in the surgical group), head growth deflection was observed among 3 patients (1.9%): 2 of 65 (3.1%) in the surgical group and 1 of 93 (1.1%) in the conservative group (Table 2). A Fisher exact test showed no statistically significant difference in deflection rates between these groups (*P* = .57). Two patients with head growth deflection had a syndromic diagnosis, including Down syndrome and neurofibromatosis type 1, and for the third patient, genetic analysis showed a pathogenic variant unrelated to craniosynostosis.

**Table 2.** Signs of Increased Intracranial Pressure

Sign	All patients	Conservatively treated patients	Surgically treated patients	*P* value^a^
Stagnation head circumference, No./total No. (%)^b^	3/158 (1.9)	1/93 (1.1)	2/65 (3.1)	.57
Papilledema, No./total No. (%)	3/209 (1.4)	1/131 (0.8)	2/78 (2.6)	.56
Preoperative papilledema, No.	NA	NA	1	NA
Postoperative papilledema, No.	NA	NA	1	NA

Abbreviation: NA, not applicable.

^a^ Fisher exact test.

^b^ Includes only patients with head circumference measures over a period of 2 or more years.

Papilledema was observed in 3 patients (1.4%): 2 of 78 (2.6%) in the surgical group and 1 of 131 (0.8%) in the conservative group (Table 2). Fisher exact tests showed no significant differences in papilledema presence between treatment groups (*P* = .56). One patient in the surgical group, who had a known syndrome (neurofibromatosis type 1) and underwent surgery, exhibited both papilledema and head growth deflection postoperatively.

### Evaluation After Head Growth Deflection or Papilledema

Among the 5 patients who exhibited signs suggestive of increased ICP, further evaluation was performed to guide management. One patient with papilledema in the conservative group underwent additional fundoscopic examinations at short intervals, with spontaneous resolution of papilledema without intervention. Another patient had preoperative papilledema and underwent magnetic resonance imaging (MRI) prior to fronto-orbital advancement, and the papilledema resolved postoperatively. One patient developed postoperative papilledema along with head growth deflection and underwent multiple MRI scans. No clinical symptoms of increased ICP or visual impairment were observed up to 6 years of age. This patient received additional follow-up because of neurofibromatosis type 1. One patient with head growth deflection in the surgical group underwent MRI, which showed no evidence of increased ICP, and no papilledema developed during follow-up until 5 years of age. Last, the patient with head growth deflection in the conservative group underwent MRI and did not develop papilledema during follow-up. After further analyses, none of the patients required surgical intervention. All 5 patients continue to be monitored according to our clinical protocol.

## Discussion

The aim of this study was to compare signs of elevated ICP in children with metopic synostosis managed surgically vs conservatively. In a cohort of 209 patients, we observed a very low prevalence of suspected increased ICP, with no differences between treatment groups. To our knowledge, this is the first study to directly compare head growth trajectories and ICP-related outcomes between surgically and conservatively managed patients with metopic synostosis, thereby addressing a key gap in the evidence base for treatment decisions.

Our longitudinal analysis suggests distinct growth trajectories depending on treatment. Conservatively managed patients showed a steady increase in head circumference SD over time, suggesting some catch-up growth within physiological range. In contrast, surgically treated patients, who initially had higher SD values, exhibited a plateau in head growth around 20 months of age. This pattern parallels previous findings in populations with craniosynostosis and suggests that surgical remodeling may prematurely arrest skull growth.^30,31,32^ Although this flattening did not correspond with increased ICP, it implies that surgery alters the natural growth potential of the trigonocephalic skull. These findings challenge the assumption that surgical correction inherently promotes better long-term cranial growth.

Children with metopic synostosis demonstrated lower head circumference SD values than population norms, consistent with prior reports that described smaller SD values among these patients.^12^ Both male and female patients exhibited reduced head circumference relative to sex- and age-adjusted norms. However, female patients demonstrated significantly higher SD values compared with male patients, indicating that although head size was reduced in both groups, the relative deviation from normative expectations was less pronounced in female patients. The surgical cohort contained a slightly larger proportion of female patients than the conservative group (31% vs 24%), which may partially account for the observation that surgical patients had higher SD values at baseline. These sex differences require further research. Severity of metopic phenotype was associated with smaller head circumference across both groups, confirming that phenotype severity is associated with head circumference. Nonetheless, previous research has shown no association between increased ICP and metopic phenotype severity, based on the Metopic Severity Score determined by CranioRate.^14^ Our findings show that patients with metopic synostosis have a smaller head circumference, which is more apparent in male patients and patients with a severe phenotype.

Only 5 of 209 patients were suspected of increased ICP based on proxy markers. These patients underwent further evaluation, which excluded the need for additional surgical intervention for all 5 patients. No delayed surgeries were performed for ICP-related indications. This finding strongly supports a very low incidence of clinically relevant ICP in single-suture metopic synostosis and demonstrates that conservative follow-up is safe when combined with structured monitoring. Reported increased ICP prevalence in the literature varies widely (0%-33%), likely due to heterogeneous diagnostic criteria and study populations.^12,13,14,15,16,17^ Studies reporting the highest rates relied on invasive ICP measurements obtained only intraoperatively or over very limited monitoring periods, methods that could result in overestimating prevalence compared with continuous invasive 24-hour monitoring. In our cohort, fundoscopy and head circumference measurements proved practical and reliable for longitudinal surveillance, whereas invasive monitoring was not indicated. Although emerging technologies such as optical coherence tomography (OCT), optic nerve sheath diameter ultrasonography, and computed tomography– or MRI-derived measures of increased ICP show promise, their use in young children remains constrained by limited feasibility, lack of robust age-specific normative data, and restricted availability or increased radiation in routine clinical practice.^14,19,33,34,35^ OCT is generally not feasible for children younger than 4 years due to lack of cooperation. This early age range represents the most clinically critical period for detecting elevated ICP in these children. Fundoscopy and head circumference therefore remain the most pragmatic tools for routine care, although their limited sensitivity may lead to underestimations.^12,19,22^

Together, these findings indicate that surgery is not required to prevent increased ICP in most cases of metopic synostosis. Conservative management is understood to decrease perioperative risks, psychological stress, and long-term costs while not increasing ICP risk. Furthermore, our results highlight that smaller head circumference and more severe phenotype do not necessarily require surgery and that surgery itself may restrict the natural trajectory of head growth. Thus, treatment decisions should be guided by individual patient characteristics and parental preferences, rather than by an assumed risk of increased ICP alone.

Finally, it is important to recognize that children with metopic synostosis are at increased risk of neurocognitive and behavioral problems.^6,7,8,9,10^ Given the very low prevalence of increased ICP, these developmental concerns are unlikely to be explained solely by increased ICP. Rather, they may reflect an intrinsic neurodevelopmental predisposition or shared genetic mechanisms.^36,37,38^ These findings indicate that surgical correction of skull shape is unlikely to address underlying developmental risks. Another hypothesis is that neurocognitive problems may result from local brain compression. Previous research, however, found no evidence of decreased cerebral perfusion in the frontal lobes of infants with metopic synostosis prior to surgery, suggesting that local brain compression is unlikely to play a major role.^39^ Nevertheless, future research is needed to clarify the potential contributions of these different hypotheses to long-term neurocognitive outcomes. Future research should compare long-term neurocognitive, ophthalmologic, and psychological outcomes between surgical and conservative cohorts, along with evaluation of head shape.^25^

### Limitations

This study has some limitations, including its nonrandomized design, reliance on noninvasive proxy measures for ICP, and low number of ICP-related events, which reduced statistical power for subgroup analyses. Invasive ICP monitoring and advanced imaging techniques such as OCT, while more sensitive, were not feasible in this pediatric cohort, potentially underestimating subclinical elevations. Furthermore, a randomized clinical trial was not acceptable to parents and the national patient association,^25^ who strongly opposed the prospect of assigning children to treatment groups by chance. As a result, treatment allocation followed clinical decision-making and parental preference rather than randomization, which introduces the possibility of selection bias and limits the ability to draw causal inferences. Due to limited subgroup sample sizes, outcomes were not compared between surgical techniques. Future research should evaluate differences between these surgical approaches. Future prospective multicenter studies, even if nonrandomized, may help to mitigate these limitations by increasing sample size, reducing center-specific bias, and improving the generalizability of findings. In addition, future research should aim to integrate emerging noninvasive modalities, systematically compare long-term neurodevelopmental outcomes between surgical and conservative management, and evaluate the cost-effectiveness of selective surgical intervention to better guide individualized treatment strategies.

## Conclusions

In this prospective cohort of patients with metopic synostosis, signs of increased ICP were rare, with no significant difference between children receiving surgical or conservative management. These findings support close clinical monitoring as a safe alternative to routine surgery for selected patients and underscore the value of individualized treatment decisions. Future studies should assess long-term outcomes and develop strategies for risk stratification to inform personalized care.

## Notes

Supplement 1. **eTable.** Linear Mixed Model Coefficients

Supplement 2.
Data Sharing Statement
