# Supplementary material for: Head Growth and Fundoscopy as Proxies for Intracranial Pressure in Metopic Synostosis Treated Surgically vs Conservatively
Source: JAMA Netw Open. 2026 Feb 24;9(2):e2559871. doi: 10.1001/jamanetworkopen.2025.59871 (PMC12933276; doi:10.1001/jamanetworkopen.2025.59871)
Supplement: Supplement 1. — eTable. Linear Mixed Model Coefficients [file jamanetwopen-e2559871-s001.pdf]

Supplementary Online Content

Tio PAE, Koehoorn EN, Clement LIF, et al. Head growth and fundoscopy as proxies for intracranial pressure in metopic synostosis treated surgically vs conservatively. *JAMA Netw Open.* 2026;9(2):e2559871. doi:10.1001/jamanetworkopen.2025.59871

eTable. Linear Mixed Model Coefficients

This supplementary material has been provided by the authors to give readers additional information about their work.

© 2026 Tio PAE et al. *JAMA Network Open.*

eTable. Linear Mixed Model Coefficients

Predictor	Estimate (β)	95% CI	p-value
Intercept	–0.62	[–0.87, –0.37]	<0.001
Age (spline 1)	0.87	[0.54, 1.21]	<0.001
Age (spline 2)	0.73	[0.29, 1.16]	0.001
Sex (Female)	0.31	[0.03, 0.60]	0.031
Treatment (Surgical)	0.37	[0.06, 0.71]	0.019
Severity (Moderate vs. Mild)	–0.22	[–0.50, 0.07]	0.145
Severity (Severe vs. Mild)	–0.39	[–0.75, –0.04]	0.030
Age (spline 1):Surgical Treatment	–1.02	[–1.52, –0.52]	<0.001
Age (spline 2):Surgical Treatment	–0.36	[–0.96, 0.24]	0.243

© 2026 Tio PAE et al. *JAMA Network Open.*
