# Supplementary material for: Head Growth and Fundoscopy as Proxies for Intracranial Pressure in Metopic Synostosis Treated Surgically vs Conservatively
Source: JAMA Netw Open. 2026 Feb 24;9(2):e2559871. doi: 10.1001/jamanetworkopen.2025.59871 (PMC12933276; doi:10.1001/jamanetworkopen.2025.59871)
Supplement: Supplement 2. — Data Sharing Statement [file jamanetwopen-e2559871-s002.pdf]

Data Sharing Statement

Tio. Head Growth and Fundoscopy as Proxies for Intracranial Pressure in Metopic Synostosis Treated Surgically vs Conservatively. *JAMA Netw Open*. Published February 24, 2026. doi:10.1001/jamanetworkopen.2025.59871

Data
Data available: No
